# Trust-Centered Design and Feasibility Evaluation of an AI-Enabled Conversational Health Tool for Sexual and Reproductive Health Among Rural Young Adults: Protocol for a Mixed Methods Study

**DOI:** 10.2196/97653

**Published:** 2026-08-06

**Authors:** Kandyce Brennan

**Affiliations:** 1 School of Nursing, University of North Carolina at Chapel Hill, Carrington Hall, Chapel Hill, NC, 27514, United States, (919) 966-4260

**Keywords:** artificial intelligence, AI, conversational agents, chatbot, sexual and reproductive health, rural health, health disparities, human-centered design, mixed methods, feasibility, digital health equity, trust, implementation science

## Abstract

**Background:**

Young adults aged 18 to 25 years in rural communities face barriers to sexual and reproductive health (SRH) information, including clinician shortages, clinic closures, and privacy concerns in close-knit communities. Many rely on online sources of varying quality; one analysis found that 40% of birth control video content is inaccurate or misleading. AI-enabled conversational health tools (chatbots) may provide scalable SRH information, but implementation may be constrained by low institutional trust, privacy concerns, and rural users’ underrepresentation in AI development. SARHAchat is an AI-enabled SRH conversational health tool developed through prior work. This protocol describes a study to co-design and evaluate SARHAchat with rural young adults, treating trust as a design input rather than a postdeployment outcome.

**Objective:**

This study aims to (1) identify multilevel determinants of trust and acceptability for AI-enabled conversational health tools among rural pregnancy-capable individuals aged 18 to 25 years in North and South Carolina; (2) co-design a SARHAchat prototype with rural stakeholders using human-centered design; and (3) evaluate its feasibility, acceptability, usability, trust, and implementation outcomes in a pilot. The goal is to develop methods that support responsible implementation of conversational AI health tools in rural and underserved communities.

**Methods:**

This protocol uses a 3-phase mixed methods design with exploratory sequential logic. In phase 1, we will conduct semistructured interviews (target n=24) with pregnancy-capable individuals aged 18 to 25 years living in Health Resources and Services Administration (HRSA)-designated rural counties in North and South Carolina to identify determinants of trust and acceptability and develop a conceptual framework. In phase 2, we will convene 5 to 8 stakeholders per session across 2 community feedback sessions to co-design and refine SARHAchat. In phase 3, we will conduct a nonrandomized mixed methods feasibility pilot with 75 pregnancy-capable individuals aged 18 to 25 years from rural counties, recruited through community-identified social media platforms.

**Results:**

This proposal was funded in February 2026. All 3 phases have been approved by the University of North Carolina at Chapel Hill Institutional Review Board (26-0669). A total of 14 participants have enrolled in phase 1. Phase 2 co-design activities are planned for August to October 2026, and phase 3 pilot recruitment is expected to begin in January 2027, with data collection concluding in October 2027. Findings are anticipated to be submitted for publication in April 2028.

**Conclusions:**

This protocol describes a feasibility study of a community-informed AI-enabled conversational SRH tool for rural young adults. By identifying trust-related design needs, refining a prototype through co-design, and generating preliminary feasibility, acceptability, usability, and implementation data, the study aims to establish trust-by-design methods that guide future effectiveness testing and may transfer to other sensitive health domains and underserved communities.

## Introduction

### Background

More than 19 million individuals of reproductive potential in the United States live in communities with limited access to comprehensive sexual and reproductive health (SRH) care [1]. Adolescents and young adults account for nearly half of the estimated 26 million incident sexually transmitted infections (STIs) occurring annually in the United States [2], and many pregnancies in this age group are unintended [3,4]. These risks are compounded in rural communities, where young adults face fewer SRH clinicians per capita, reduced continuity of care, and privacy constraints inherent to close-knit communities [1,5-7].

When clinical care is inaccessible, rural young adults increasingly seek SRH information through digital platforms. In one analysis of YouTube birth control videos, roughly 40% of reproductive health content was identified as inaccurate or misleading [8]. This problem is compounded by health data poverty: the systematic underrepresentation of rural populations in the datasets used to train AI systems. This data gap degrades the performance and cultural relevance of digital health tools for the communities that need them the most [9,10]. At the same time, rural communities often exhibit lower baseline trust in institutional systems and emerging technologies, a skepticism that is often historically justified, yet existing AI health tool development has overwhelmingly treated trust as a postdeployment outcome to measure rather than as a foundational design input [11,12].

### The Opportunity: AI Conversational Health Tools and Their Translational Barriers

AI-enabled conversational health tools, commonly referred to as chatbots, hold promise for addressing rural SRH information gaps. These tools can deliver personalized, confidential, on-demand health guidance at scale without requiring a clinical appointment, transportation, or disclosure of one’s identity to community members [9,13-17]. For sensitive health domains in which stigma and social judgment may inhibit care seeking, this combination of accessibility and anonymity is particularly valuable.

However, the translational gap between promising AI technology and meaningful engagement in rural communities remains wide. Existing conversational AI tools for SRH have been developed and tested in predominantly urban samples, with minimal attention to rural users’ communication preferences, technological constraints, literacy levels, or community values [9,18]. The result is a cycle of poor fit: tools designed without rural communities generate low engagement, which is misread as disinterest, which further excludes rural users from digital health innovation [12]. Addressing this cycle of poor fit and ensuring appropriate data representation within AI digital health tools requires a co-design process that begins with the community rather than ending with community feedback.

### Prior Work

SARHAchat is an AI-enabled conversational health tool designed to increase access to trustworthy, evidence-based SRH information through personalized, on-demand guidance and confidential decisional support. A beta prototype was developed and piloted in prior work, with initial pilot data demonstrating acceptable usability in a general adult sample (ages of 18‐44 years) [14,19]. That pilot also revealed important limitations: rural participants were underrepresented, and users expressed a desire for more personalized information. These findings pointed directly to the need for a systematic, community-engaged redesign process in which rural young adults are active collaborators.

### Conceptual and Theoretical Grounding

This research is grounded in the socioecological model [20], which situates health behavior at the individual, interpersonal, organizational, community, policy, and societal levels. This grounding frames the design of interventions around users’ values and needs. Human-centered design (HCD) and community-based participatory research principles [21,22] guide the co-design methodology, which positions community members as partners throughout the research process rather than as consultants at the end. Implementation science, specifically the reach, effectiveness, adoption, implementation, and maintenance (RE-AIM) framework [23], structures the feasibility evaluation, ensuring that implementation is documented systematically from the earliest stages of piloting.

Trust is treated in this research as a multilevel, modifiable construct and not a fixed community characteristic. Individual-level trust involves beliefs about AI accuracy, data privacy, and response quality. Interpersonal and community-level trust encompasses social norms around technology use for health matters and the influence of peer and partner relationships on SRH decision-making [24]. Structural trust reflects historical and ongoing relationships between communities and health care institutions. This framing draws on an emerging evidence base identifying trust as both a key moderator of conversational AI engagement and an addressable design-level variable [17]. Embedding trust as a design principle rather than solely measuring it as an outcome after deployment is the central innovation of the proposed research.

Our study leverages HCD to embed empirically derived determinants of trust into an AI-enabled conversational SRH tool. Our overarching goal is to develop and evaluate an enhanced, community-informed version of SARHAchat co-designed with rural communities and grounded in these determinants of trust. This objective will be achieved through the following 3 phases. Phase 1 aims to characterize the multilevel, trust-related determinants that shape the acceptability of AI-enabled conversational health tools among pregnancy-capable (assigned female at birth) young adults aged 18 to 25 years in rural communities and develop a conceptual framework of trust determinants. Phase 2 aims to co-design and adapt an enhanced, community-informed SARHAchat prototype with rural stakeholders using an HCD approach. Phase 3 aims to evaluate the enhanced prototype’s feasibility, acceptability, usability, trust, and early implementation outcomes through a community-based pilot. This project aims to reduce disparities in access to SRH information and advance innovative, community-informed, technology-based solutions.

## Methods

### Overview of Research Design

The proposed research uses a 3-phase design that moves deliberately from qualitative inquiry to participatory co-design to feasibility piloting of an AI-enabled conversational agent (SARHAchat). This is a multiphase mixed methods design in which an exploratory sequential logic links the phases. Qualitative findings from phase 1 inform the co-design requirements for phase 2, which in turn produce the intervention evaluated in phase 3. An explanatory sequential design within phase 3 integrates quantitative feasibility outcomes with qualitative interview data through a RE-AIM–organized joint display. This phased study reflects the project’s translational logic: community-derived determinants of trust must inform prototype development before the tool is evaluated, and implementation strategies must be codeveloped with communities before they are tested.

### Description of the Chatbot

SARHAchat (which stands for “Sexual and Reproductive Health Assistant Chatbot”) is an AI-enabled conversational agent developed through previously funded research [14,19]. SARHAchat features include user-guided question-and-answer sessions or a predefined 5-stage conversational flow (intent gathering, preference screening, basic health screening, method recommendation, and a downloadable summary to support subsequent clinician interactions and prescription workflows) managed by a stage tracker that monitors the conversational state and transitions. SARHAchat is built on GPT-4 (OpenAI) accessed via the vendor API and hosted on institutionally approved Amazon Web Services (AWS) infrastructure. Contraceptive and SRH guidance is not generated solely from the model’s parametric knowledge; a retrieval-augmented generation layer grounds each response in a curated, expert-maintained knowledge base of current clinical guidance (eg, the 2024 Centers for Disease Control and Prevention US Medical Eligibility Criteria for Contraceptive Use and selected practice recommendations and the Food and Drug Administration product labeling). A structured reasoning chain co-designed with health care experts is combined with a “thought injection,” which is a prompting technique in which predefined expert reasoning steps are inserted into the model’s prompt to steer it toward guideline-concordant, contextually appropriate recommendations while preserving natural conversational flow. Retrieval grounding and rule-based checks verify each recommendation against the user’s screening responses and stated preferences, together forming the system’s accuracy safeguards and complementing the safety guardrails described in the following section. A generative, multimodal interface pairs text with visual aids and a downloadable PDF summary to enhance comprehension and clinician handoff.

### Clinical Safety and High-Risk Query Response Protocol

**Figure 1.**
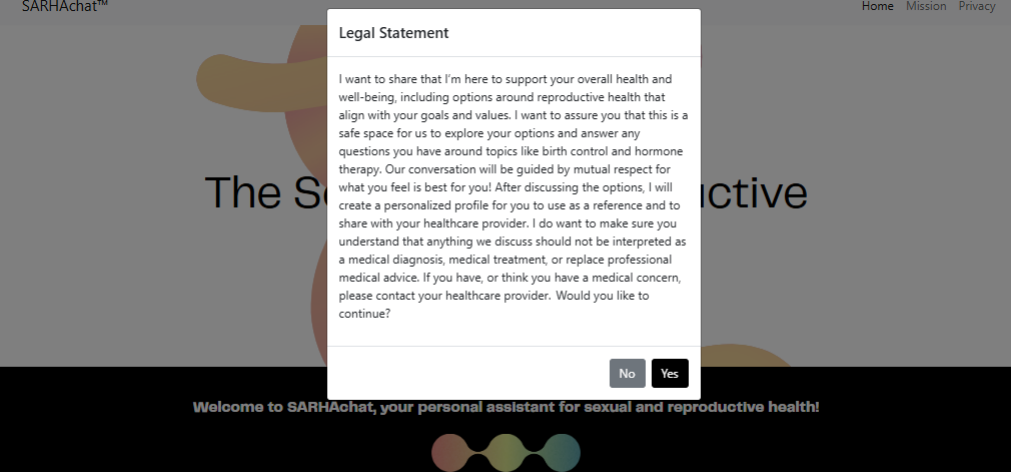
SARHAchat scope disclosure.

SARHAchat provides general, evidence-based SRH information and decision support. It is not a diagnostic tool, emergency service, prescribing platform, crisis response system, or substitute for care from a licensed clinician. The scope of SARHAchat is stated at the start of use and reinforced when users raise urgent symptoms, sexual or partner violence, mental health crises, or other high-risk concerns (Figure 1). Programmed safety guardrails identify prompts involving urgent clinical needs (eg, STI symptoms or exposure, emergency contraception, or abortion-related concerns) and safety concerns (eg, sexual coercion or assault, intimate partner violence, human trafficking, or mental health crises). When such content is detected, SARHAchat responds with supportive language, clarifies that it cannot assess or diagnose, and refers the user to a clinician or appropriate resource (eg, the 988 Suicide & Crisis Lifeline, Substance Abuse and Mental Health Services Administration National Helpline, National Sexual Assault Hotline, and National Domestic Violence Hotline). These guardrails complement the system’s additional safety measures, including retrieval-augmented generation, which grounds responses in verified clinical guidelines to reduce hallucinations, whereas additional checks ensure that each recommendation accounts for health screening results and stated preferences. For pregnancy, contraception, emergency contraception, STI testing or treatment, and abortion-related questions, SARHAchat offers general educational information and directs users to qualified resources such as a clinician, a local health department, or Planned Parenthood. Because the study team is not providing clinical care, referrals are informational and safety-oriented rather than related to clinical management. Participants are encouraged to seek qualified professionals for individualized evaluation, diagnosis, treatment, or follow-up and are reminded that they may stop using SARHAchat or withdraw at any time without penalty or loss of access to educational and safety resources.

### Phase 1: Identifying Multilevel Determinants of Trust and Acceptability

#### Rationale

Understanding trust formation in human-AI health interactions requires examining individual, interpersonal, and structural factors that shape technology acceptance in communities with legitimate reasons for institutional skepticism. Preliminary pilot data from our initial SARHAchat prototype demonstrated acceptability among a general adult sample but revealed underrepresentation of rural participants and a desire among users for more personalized, contextually sensitive information [14]. Evaluations of AI conversational health tools in health care contexts more broadly document user satisfaction when tools are properly implemented [9,17], yet significant challenges remain in translating these findings to rural communities. Acceptability in these settings is shaped by factors including data privacy concerns, perceived response accuracy, cultural appropriateness, and the relationship contexts in which health decisions are made [9,15,17]. These determinants must be identified before design decisions are made.

#### Proposed Approach

We will recruit a target of 24 pregnancy-capable individuals aged 18 to 25 years residing in rural designated counties in North Carolina (NC) and South Carolina (SC) as defined by the Health Resources and Services Administration (HRSA) [25], with an a priori stopping rule assessed in waves. On the basis of the work by Hennink and Kaiser [26], we anticipate reaching saturation with between 14 and 32 interviews and will extend recruitment to a maximum of 32 if needed. Purposive sampling will be based on race and ethnicity, sexual identity, and state to ensure diverse representation of the target population. Thematic saturation will be assessed in waves and will stop when 3 or more consecutive interviews add no new codes. Recruitment will combine social media geofencing targeting HRSA-designated rural counties with community-based outreach through established community partners, an approach that proved effective in our initial SARHAchat pilot and is adapted here to specifically target rural counties.

Interviews will be conducted virtually and will explore current health information–seeking behaviors and preferences; relationship and social contexts shaping SRH decision-making; technology use and self-reported digital health literacy; acceptability criteria and conditions that would increase or decrease willingness to engage with AI health tools; specific trust concerns and perceived benefits; and rural-specific influences, including device availability, privacy dynamics, and health care access patterns.

#### Expected Outcomes

Phase 1 will produce a preliminary community-informed conceptual framework specifying how individual, interpersonal or community, and structural factors shape trust in conversational AI within the SRH domain among rural young adults. We anticipate identifying 3 to 5 critical trust dimensions, each with specific, actionable design requirements. These findings will serve as the evidence base for phase 2 co-design activities and will identify trust as a modifiable implementation determinant.

### Phase 2: Co-Design and Adaptation of SARHAchat Using HCD

#### Rationale

Phase 1 will identify the determinants of trust and acceptability to be addressed in the tool design; phase 2 will translate those determinants into tool features and functionality. Many conversational AI digital health tools are designed for high-resource, often urban communities, creating a poor fit for rural users [10-12,17]. Through structured, iterative co-design, we will ensure that community members directly shape the prototype, preventing a common cycle in which poorly adapted tools lead to low engagement, low engagement is misinterpreted as disinterest, and rural communities are further excluded from digital health innovation [18].

#### Proposed Approach

##### Overview

We propose convening 5 to 8 stakeholders per session for 2 structured, iterative community feedback sessions. Stakeholders will represent community end users reflecting phase 1 demographics, peer health education and community support, and community health workers. Participants may contribute expertise across multiple areas.

##### Session 1: Feedback and Feature Mapping

Stakeholders will interact directly with the current SARHAchat prototype and provide structured feedback on usability, cultural responsiveness, acceptability, and data privacy. An information ecosystem mapping exercise will position SARHAchat within participants’ existing digital health-seeking behaviors, and a platform preference assessment will identify which social media channels to target in phase 3. Participant feedback will be synthesized into a set of targeted design modifications, each directly linked to specific trust and acceptability barriers from phase 1.

##### Session 2: Iteration, Validation, and Implementation Refinement

A mixed group of returning and new stakeholders will review the updated prototype incorporating session 1 modifications. New participants will receive an orientation summarizing prior findings. The session will reassess the core trust and usability domains, validate that the concerns raised in session 1 were adequately addressed, and further refine digital implementation strategies for the social media deployment planned for phase 3. Returning participants will provide continuity; new participants will identify areas of convergence and remaining divergence. Qualitative data across both sessions, facilitator notes, and audio transcripts will be thematically coded to document iterative refinement and produce a trust-by-design mapping document linking each design decision to the community-derived trust determinants that motivated it.

### Expected Outcomes

Phase 2 will produce a community-informed SARHAchat prototype with documented trust-building and acceptability features, a design decision log, and a preliminary set of digital implementation strategies. This community-informed prototype constitutes the intervention to be evaluated in phase 3.

### Phase 3: Feasibility Pilot of the Enhanced SARHAchat Prototype

#### Rationale

Phases 1 and 2 will identify trust factors and co-design a community-informed prototype, but a translational gap remains in understanding how the tool performs in real-world rural contexts, specifically whether participants can be successfully recruited to it and whether it can be used and sustained via social media platforms that rural young adults already use.

#### Proposed Approach

We propose a nonrandomized feasibility pilot that deploys the community-informed SARHAchat prototype on 2 social media platforms identified by the target population (ages of 18‐25 years; HRSA-designated rural NC and SC counties) during phases 1 and 2. This “meet people where they are” implementation strategy departs from traditional clinic-based digital health delivery and tests a potentially more equitable and scalable approach to reaching rural young adults.

A total of 75 pregnancy-capable individuals aged 18 to 25 years residing in HRSA-designated rural counties will be recruited via social media geofencing, accounting for a projected 20% attrition rate and yielding a target of 60 completers, which is consistent with published recommendations for digital health feasibility studies [27]. To achieve this within a 16-week recruitment window, the required enrollment rate is approximately 4.7 participants per week. Prospective monitoring will trigger adaptive recruitment if fewer than 30 participants have been enrolled by week 8.

**Figure 2.**
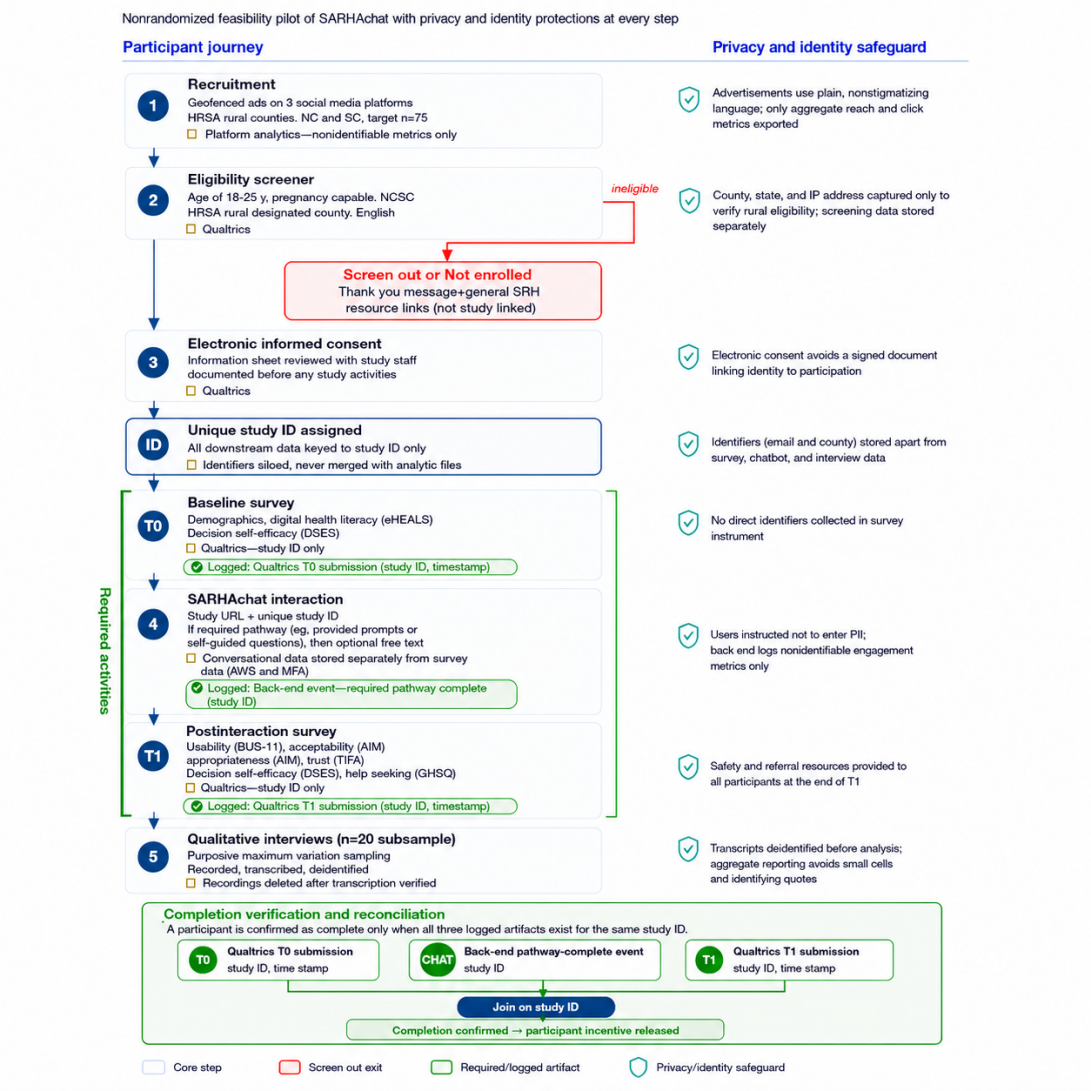
Flow for participant activities for the phase 3 feasibility pilot. AIM: Acceptability of Intervention Measure; AWS: Amazon Web Services; BUS-11: Bot Usability Scale; DSES: Decision Self-Efficacy Scale; eHEALS: eHealth Literacy Scale; GHSQ: General Help Seeking Questionnaire; HRSA: Health Resources and Services Administration; IAM: Intervention Appropriateness Measure; MFA: multifactor authentication; NC: North Carolina; PII: personally identifiable information; SC: South Carolina; SRH: sexual and reproductive health; T0: baseline; T1: after interaction with the chatbot; TPA: Trust Between People and Automation scale.

Eligible participants will complete an electronic informed consent form and a baseline survey (T0; digital health literacy, decision self-efficacy, and demographics), immediately engage with the enhanced SARHAchat prototype to complete one pathway (eg, contraception consultation or question-and-answer session) with a guided prompt or their own questions, and then complete a postinteraction survey (T1; usability, acceptability, appropriateness, trust, and decision self-efficacy). A purposively selected subsample of 20 participants will complete semistructured interviews to provide explanatory qualitative depth (Figure 2).

#### Data Privacy and Confidentiality

Research staff are responsible for maintaining the confidentiality of study participants. Identifiable contact information collected for screening, consent coordination, scheduling, compensation, or optional interview follow-up will be stored separately from survey responses, interview data, and SARHAchat interaction data. Identifiable information will be used only for approved study operations and will not be merged with analytic datasets. Participants will be instructed not to enter personally identifiable information into the chatbot, and any conversational data that contain such information will be removed by study staff. The conversational data are accessible only to approved study staff and stored on the AWS platform protected by multifactor authentication. Conversational data are stored for the duration of the pilot study and data analysis.

#### Data Analytical Plan

##### Phase 1

Qualitative data (ie, health information–seeking behaviors, determinants of trust, and opinions regarding chatbot development) will be transcribed and loaded into the qualitative analysis software ATLAS.ti (ATLAS.ti Scientific Software Development GmbH) for analysis. A hybrid deductive-inductive approach will be used, including initial code development based on prior literature and relevant conceptual models, which will be refined through iterative review of transcripts [28]. Data that do not fit the initial coding structure will be coded inductively; discussed by the analytic team; and incorporated as new codes, subthemes, or themes as appropriate. Analysis will explore variation by race and ethnicity and geographic location, with particular attention to rural-specific factors absent from urban-centric prior literature [17,18,22].

##### Phase 2

Across both sessions, data will include facilitator notes, audio-recorded and transcribed group discussions, and design artifacts generated during structured activities (information ecosystem maps and implementation insights). Transcripts and notes will be analyzed using rapid analysis with structured summary templates [29] to enable timely synthesis between sessions and inform iterative SARHAchat refinement. Summaries will be charted into comparison matrices to identify areas of convergence (validation) and divergence (further adaptation needs) across sessions 1 and 2. Each design modification will be recorded in a decision log that links participant feedback to the specific trust and acceptability determinants from phase 1 or to newly emerged determinants from sessions, producing a trust-by-design mapping and an analytical audit trail. Credibility will be supported via member checking as returning session 2 participants will validate whether session 1 concerns were addressed [30].

##### Phase 3

*Quantitative* data include demographic and socioeconomic characteristics; baseline digital health literacy (eHealth Literacy Scale) [31]; and implementation, usability, acceptability, appropriateness, and trust outcomes (Bot Usability Scale [32], Acceptability of Intervention Measure [33], Intervention Appropriateness Measure [33], and Trust Between People and Automation scale [34]) supplemented by back-end engagement analytics. Additionally, the Decision Self-Efficacy Scale (DSES) [35] will be measured at T0 and T1, and an adapted General Help Seeking Questionnaire (GHSQ) [36] will be administered at T1 to assess exploratory effectiveness signals. Due to the pilot sample size (n=75), we will perform descriptive analyses to present participants’ characteristics (eg, demographics, digital health literacy, and geographic location) and user experiences (eg, acceptability, usability, appropriateness, and trust). We will observe potential similarities and differences in implementation and use outcomes across participant characteristics (eg, race and ethnicity and geographic location), which we will interpret as hypothesis generating given the sample size. Usability data gathered by the SARHAchat dashboard will describe user activities such as session timing, completion and retention rates, and number of conversational exchanges. Feasibility outcomes will be organized within the RE-AIM framework against prespecified progression thresholds (Table 1). Consistent with the pilot’s feasibility scope, effectiveness through the DSES and GHSQ will be evaluated as exploratory and not a test of efficacy. We will report the mean within-person change in DSES and mean GHSQ. From the pilot sample, 20 participants will be purposively selected for semistructured interviews using maximum variation in engagement level, feasibility outcomes (including threshold missing cases), race and ethnicity, and geographic location to explain the range of quantitative findings. *Qualitative* data (ie, opinions and experiences regarding SARHAchat usability, acceptability, and trust) will be transcribed and analyzed using rapid thematic analysis with structured summary templates [28] and charted into a RE-AIM–aligned matrix [23,37,38]. A hybrid deductive-inductive codebook [28] will use RE-AIM domains as the deductive scaffold, with inductive codes capturing emergent themes. Integration of quantitative and qualitative data will be achieved through a RE-AIM–organized joint display [37,38] pairing each domain’s quantitative result with its qualitative explanatory theme to produce actionable meta-inferences (Multimedia Appendix 1). These meta-inferences will directly inform the progression decision, distinguishing modifiable design-level barriers from structural ones and guiding whether tool refinement or revision of the implementation strategy is needed [39].

**Table 1.** Proposed feasibility outcomes, instruments, time points, and prespecified progression thresholds for aim 2, organized within the reach, effectiveness, adoption, implementation, and maintenance (RE-AIM) framework.

RE-AIM domain	Outcome and measure	Time point	Prespecified threshold
Reach	Enrollment rate and consent rate	16-wk recruitment window	≥60% of target (n≥45 enrolled)
Effectiveness	DSES^a^ [35] and adapted GHSQ^b^ [36]	DSES: T0^c^ and T1^d^; GHSQ: T1	Exploratory; no progression threshold; mean T0-T1 DSES change and T1 GHSQ
Adoption or engagement	Proportion completing the chatbot pathway among all enrolled	After interaction	70% or more of the participants completing a session
Implementation—usability	BUS-11^e^ [32]	T1	Mean BUS-11>4
Implementation—acceptability	AIM^f^ [33]	T1	Mean AIM>4
Implementation—appropriateness and trust	IAM^g^ [33] and TPA^h^ [34]	T1	Mean IAM>3; mean distrust<4; mean trust>4
Implementation—retention	Proportion completing the T1 survey after completing the chatbot pathway	T1	70% or more of the participants completing a session
Maintenance	Tool uptime (percentage of time that the chatbot is accessible) and reported error or failure rates	After T1	≥80% uptime and ≤20% error or failure rate

a DSES: Decision Self-Efficacy Scale.

b GHSQ: General Help Seeking Questionnaire.

c T0: baseline.

d T1: after interaction with the chatbot.

e BUS-11: Bot Usability Scale.

f AIM: Acceptability of Intervention Measure.

g IAM: Intervention Appropriateness Measure.

h TPA: Trust Between People and Automation scale.

### Data Integrity, Missing Data, and Handling of Incomplete or Failed Interactions

A participant is classified as a completer only when the Qualtrics (Qualtrics International Inc) T0 submission, the back-end pathway-complete event, and the Qualtrics T1 submission all exist for the same study ID (Figure 2). All thresholds are calculated against the number enrolled except for retention, which is calculated among pathway completers to isolate postengagement attrition. Geographic eligibility will be verified by comparing self-reported county with the IP address captured by the Qualtrics screener; discordant or anonymized (eg, virtual private network) locations will be manually reviewed and screened out if unresolved via a brief verification contact. Duplicate enrollment and potential bot submissions will be addressed through Qualtrics duplicate submission and bot controls. Only the first consented record will be retained. IP addresses will remain in the siloed screening dataset, used solely for eligibility verification and duplicate detection, and will be deleted when recruitment closes.

Participants without a pathway-complete event within 2 days of T0 will be counted as noncompleters for the adoption threshold, and their partial engagement (number of exchanges and last conversational stage reached) will be described to identify points of disengagement. Back-end logs will distinguish verified technical failures (system unavailability, response generation errors, and loss of session state) from user abandonment and from safety guardrail activations, which reflect intended behavior and are reported separately. Technical failures contribute to the maintenance error rate (≤20%), and affected participants may reattempt using their original study ID, with adoption status determined via eventual pathway completion. Participants whose session is interrupted by a technical failure may resume via their original study ID within 7 days; sessions not completed within this window are classified as noncompleters. Given the pilot’s descriptive scope, analyses will follow an available-case approach. Scale scores will be computed when at least 80% of items are answered, DSES change will be limited to participants who complete both T0 and T1, and baseline characteristics of completers and noncompleters will be compared descriptively.

### Progression Criteria

The prespecified criteria for advancing to an effectiveness trial are as follows: reach of 60% or more; retention of 70% or more; mean Bot Usability Scale, Acceptability of Intervention Measure, and Intervention Appropriateness Measure scores above 4; mean distrust score below 4; mean trust score above 4; and 70% or more of the participants completing the chat session and T1. Effectiveness measures (DSES and GHSQ) are exploratory, intended to inform the design of any future effectiveness trial, and are not progression metrics. If the criteria are not met, qualitative findings will be used to determine whether barriers are modifiable at the design level or structural, distinguishing between tool adaptation and revision of the implementation strategy as the appropriate response.

### Ethical Considerations

At the time of manuscript submission, all 3 phases of this study had been approved by the Institutional Review Board of the University of North Carolina at Chapel Hill (26-0669).

All participants will provide informed consent before any study activities begin. Individuals responding to social media recruitment will be directed to a Qualtrics screening link. For phase 1 interviews, eligible participants will be contacted by the research team via email restating the purpose of the study, with a link to self-schedule an interview conducted using an institutional Zoom (Zoom Video Communications) account. A trained member of the study team will confirm eligibility; describe the study’s purpose, procedures, risks, and benefits; and obtain verbal consent prior to the interview. For phase 2 feedback sessions and phase 3 chatbot interaction and surveys, consent will be obtained electronically via Qualtrics, where participants will review an information sheet detailing the study’s purpose, procedures, risks, and benefits and provide documented consent before any study activities begin. Data collection will follow a minimization approach: county and state of residence will be collected only to confirm rural eligibility, and each participant will be assigned a unique study ID. Identifiers (email address, county, and IP address) will be stored separately from research data and will never be merged with transcript or analytic files. Audio recordings will be transcribed and then permanently deleted following verification of transcript accuracy, and transcripts will be deidentified prior to analysis. Electronic data will be stored on secure, password-protected university servers with access restricted to authorized members of the research team, and SARHAchat back-end data will be stored on institutionally approved, secure AWS infrastructure with multifactor authentication.

Participants will access SARHAchat through a study URL and a unique study ID. Before engaging with the chatbot, participants must acknowledge a safety statement explaining that the chatbot provides informational support only and is not a substitute for clinical care and that they should consult a health care professional for evaluation, diagnosis, or treatment as needed (Figure 1). Participants will be instructed not to enter names, addresses, phone numbers, exact locations, names of partners or family members, or other directly identifying information into the chatbot. To further reduce unnecessary disclosure, phase 3 will offer 2 modes of engagement: guided prompts that allow participants to explore common SRH topics without typing personal questions and optional free-text entry for participants who wish to ask their own questions. High-risk prompts, including those involving urgent symptoms, sexual coercion, partner violence, or emotional crises, will trigger predefined safety and referral responses directing users to emergency, clinical, or crisis services as appropriate.

Participants will be compensated for their time in recognition of their contribution to the study. Compensation is structured by study phase: US $50 for phase 1 interviews, US $100 for phase 2 feedback session participation, and US $45 for phase 3 chatbot interaction and T1 survey completion, with an additional US $50 for those who choose to participate in a follow-up phase 3 interview.

## Results

The proposed research was funded in February 2026. Phase 1 semistructured interviews are scheduled to commence in May 2026; phase 2 community co-design sessions are planned for August 2026 to October 2026; and phase 3 pilot recruitment is projected to start in January 2027, with data collection concluding by October 2027. Full study results are anticipated to be submitted for publication in April 2028 (Table 2). At the time of protocol submission, a total of 14 participants have been enrolled in phase 1.

**Table 2.** Projected timeline for proposed research activities and primary outputs.

Activity	Year 1 (2026)	Year 2 (2027)	Primary output
Protocol finalization and study launch	Quarter 1-quarter 2	—^a^	Approved protocol
Phase 1: semistructured interviews (target: n=24; range 14-32) and rapid thematic analysis	Quarter 2-quarter 3	—	Conceptual framework
Phase 2: co-design sessions and prototype revision	Quarter 3-quarter 4	—	Community-informed SARHAchat version 2
Phase 3a: pilot recruitment and data collection (16 wk) and pilot participant interviews (n=20)	—	Quarter 1-quarter 3	Feasibility dataset
Phase 3b: integrated analysis	—	Quarter 3-quarter 4	Feasibility manuscript

a Not applicable.

## Discussion

### Significance and Principal Contributions

This study represents a systematic effort to embed trust as a foundational design principle throughout the full development and community piloting cycle of an AI-enabled conversational health tool targeting rural young adults in the SRH domain. By generating a conceptual framework of rural trust determinants, producing community-derived design and implementation strategies, and piloting a social media–based deployment strategy, this research aims to inform responsible conversational AI design for this population and generate hypotheses that may transfer to other sensitive health domains and underserved communities.

The sequential design, moving from qualitative inquiry to participatory co-design to feasibility piloting, reflects a deliberate translational science logic grounded in implementation science. Each phase directly informs the next. This architecture seeks to avoid the common failure mode in AI health tool development in which tools designed without adequate community input generate poor engagement and this poor engagement is misattributed to community disinterest rather than design inadequacy [12,13,23].

### Innovation

This research reframes trust from a postdeployment outcome to a design input, operationalizing it as a multilevel construct with community-derived design requirements. To our knowledge, an explicit trust-by-design mapping of this kind has not been described in the SRH conversational AI literature, and the methods developed in this study may be adaptable to other sensitive health domains. The study also aims to demonstrate a community-informed process for developing conversational AI health tools that future research could draw on and adapt.

### Limitations

Phase 3 is not powered to detect the efficacy of SARHAchat; it is designed as a feasibility and signal detection study consistent with established recommendations for pilot work [27,39], and the findings will generate hypotheses about effectiveness. Recruitment via social media geofencing may introduce selection bias toward individuals with higher baseline digital literacy and existing social media access; the eHealth Literacy Scale administered at baseline will characterize this variability, and qualitative components will probe access as a barrier. The pilot does not measure long-term behavior change based on intervention use. Geographic restriction to NC and SC limits immediate generalizability, although the HRSA-based rural sampling strategy and emphasis on community-informed design principles are intended to maximize transferability within the rural US context.

### Future Directions and Impact

The primary contribution of this study is feasibility evidence: whether participants can be successfully recruited to and engage with a community-informed, trust-centered conversational agent and whether it can be sustained on the social media platforms that rural young adults already use. If the pilot meets progression criteria, the resulting feasibility estimates, refined implementation strategies, and exploratory signal detection data would inform the outcome selection and design for a subsequent effectiveness trial. Any criteria that are not met would indicate whether tool refinements or implementation strategy revisions are needed before an effectiveness trial is warranted. The phase 1 conceptual framework and the phase 2 and 3 community-informed methods aim to add to the emerging literature on responsible, equity-centered conversational AI design.

### Conclusions

This study describes a community-engaged research program designed to address a persistent equity gap in digital health: the limited treatment of trust as a design principle in AI-enabled health tools developed for rural and underserved communities. By generating a conceptual framework of trust determinants, a community-informed prototype, and feasibility data grounded in the lived experiences of rural young adults, this work aims to support more responsible development and implementation of conversational AI for communities facing persistent barriers to SRH care.

## Supplementary material

10.2196/97653 Multimedia Appendix 1 Reach, effectiveness, adoption, implementation, and maintenance (RE-AIM) framework joint display.

10.2196/97653 Peer Review Report 1 Peer review report from UNC (University of North Carolina at Chapel Hill) Building Interdisciplinary Research Careers in Women’s Health (BIRCWH) Program Review Committee.

10.2196/97653 Peer Review Report 2 Peer review report from UNC (University of North Carolina at Chapel Hill) Building Interdisciplinary Research Careers in Women’s Health (BIRCWH) Program Review Committee.

## Abbreviations

AWS: Amazon Web Services
DSES: Decision Self-Efficacy Scale
GHSQ: General Help Seeking Questionnaire
HCD: human-centered design
HRSA: Health Resources and Services Administration
NC: North Carolina
RE-AIM: reach, effectiveness, adoption, implementation, and maintenance
SARHAchat: Sexual and Reproductive Health Assistant Chatbot
SC: South Carolina
SRH: sexual and reproductive health
STI: sexually transmitted infection
