# Supplementary material for: Trust-Centered Design and Feasibility Evaluation of an AI-Enabled Conversational Health Tool for Sexual and Reproductive Health Among Rural Young Adults: Protocol for a Mixed Methods Study
Source: JMIR Res Protoc. 2026 Aug 6;15:e97653. doi: 10.2196/97653 (PMC13446796; doi:10.2196/97653)
Supplement: Supplementary file 1 [file resprot-v15-e97653-s002.pdf]

UNC BIRCWH REVIEW TEMPLATE

Reviewer Name: Reviewer 1

Candidate Name: Kandyce Brennan

Scoring Criteria

- Please use a separate template for each candidate you review.
- ***We will remove your name from this review prior to sharing it with the candidate.***
- We will use the NIH 9-point scale (no decimals).
    1. Exceptionally strong with essentially no weaknesses
    2. Extremely strong with negligible weaknesses
    3. Very strong with only some minor weaknesses [a]
    4. Strong, but with numerous minor weaknesses
    5. Strong, but with at least one moderate weakness [b]
    6. Some strengths, but also some moderate weaknesses
    7. Some strengths, but with at least one major weakness [c]
    8. A few strengths and a few major weaknesses
    9. Very few strengths and numerous major weaknesses
- Weaknesses are defined as follows:
    a. Minor weakness: An easily addressable weakness that does not substantially lessen impact
    b. Moderate weakness: A weakness that lessens impact
    c. Major weakness: A weakness that severely limits impact

Overall Impact

Provide an overall impact score reflecting your assessment of the likelihood for the candidate to initiate a strong research program, in consideration of the five scored review criteria (below) and the following program priorities:

- **BIRCWH program priorities:** Mentor and train early career investigators to become independent interdisciplinary translational science researchers who study high priority areas in women's health, explore biological sex influences on health and disease, and incorporate research strategies to diminish health disparities for women across the lifespan.

Overall Impact Score: 3: Very strong with only some minor weaknesses

Overall Impact Summary: Nurse Brennan is a midwife, nurse educator and researcher. She proposes a mixed methods research, human-centered design (HCD), and implementation science through training and mentorship from an excellent interdisciplinary team with Artificial intelligence (AI) conversational health tools ("chatbots") regarding SRH in a rural population aged 18-24. AI conversational health tool, SARHAchat prototype is already made. This work offers a promising and scalable strategy to bridge these gaps by expanding access to SRH information. She will study the potential real-world impact depends on overcoming key translational barriers to implementation, including trustworthiness, acceptability, accessibility, and sustained engagement, particularly in rural and underserved communities (e.g., mistrust of AI, privacy concerns, literacy barriers. This is a highly competitive application with strong alignment to BIRCWH priorities, a well-qualified candidate, and an innovative, equity-focused research plan. The mentorship and institutional support are excellent. Minor

refinements could strengthen feasibility and scalability arguments. Other members of the committee noted that the scales used for contraception in the AI tool had some flaws dampening enthusiasm.

Recommendation: Discuss in study section.

Scored Review Criteria

Please provide a separate score for each criterion.

1. Candidate Background and Research Training

Instructions Provided to Applicants: *A recent Biosketch should be uploaded into the application portal. Use updated format dated September 2024.*

Guiding Questions for Reviewers:

- Evaluate the developmental stage of the candidate. Do they have some research "traction" while still having room to benefit from additional focused training?
- Do the candidate's career goals align with the K12 program?

Score: 1: Exceptionally strong with essentially no weaknesses

Strengths: *Supported by a Society of Family Planning"Changemaker" early career fellowship, my work focuses on developing AI-enabled tools to improve patientcentered. SRH care in rural and underserved populations. 6 publications (SRH and AI ethics)*

Weaknesses: n/a

2. Candidate Career Development Plan

Instructions Provided to Applicants: *Clearly identify your training goals during the K12 program and explain how each goal aligns with your current research project and long-term career objectives. Be specific in describing how the proposed training activities will directly support your transition to becoming an independent researcher. Where appropriate, including expected timelines and completion dates for specific activities.*

Guiding Questions for Reviewers:

- Is there sufficient rationale for training?
- Does the training plan align with long term goals, i.e. learning skills that will serve to define a new niche for the candidate?
- Does the career development plan include content on community engagement and/or health disparities training?

Score: 3: Very strong with only some minor weaknesses

Strengths: Appropriate for her long term goals. Well thought out and correlates with research project.

Weaknesses: Seems heavy on course work (are all courses needed) in addition to completing aims—overly ambitious? Metrics for R01 readiness not given

3. Proposed Research Project

Instructions Provided to Applicants: *This project serves as the scholar's primary research effort. It is expected to generate most or all of the preliminary data needed for an individual K- or R-equivalent grant submission by the end of the second year of support. This plan should address key components such as the significance, innovation, and approach of the proposed project. Organize this section according to the following subsections:*

I. *Specific Aims (maximum 1 page)*

II. *Research Strategy (maximum 4 pages):*

   a. *Significance: Describe the importance of the problem, how it will advance the field, and the potential impact of your research.*
   b. *Innovation: Explain how your research challenges existing paradigms or develops novel technologies or approaches.*
   c. *Approach: Detail the experiments you will conduct to achieve each aim organized around those aims. Include your rationale for the hypothesis, expected outcomes, and alternative approaches.*

Guiding Questions for Reviewers:

- Does the research plan describe the significance and innovation potential of the research to advance women's health?
- Does the research plan serve as a foundation for future research?
- Does the research plan describe a project that will generate preliminary data for the next step of grant submission within the 2-to-3-year proposed timeframe?
- Does the scope of the research project align with the proposed timeline?

Score: 4: Strong, but with numerous minor weaknesses

Strengths: Highly significant problem to women's health. The demographic being targeted 18-24 would be amenable to using chatbots/tech. Ai chat bot is innovative and the topics of trust, accuracy, privacy, and accessibility are prioritized. Use of AI conversational health tool, SARHAchat, prototype is already developed. Addresses health care poverty. Project is feasible and aims are not interdependent. Diverse stakeholders will beta test the chatbot.

Weaknesses: Needs strong plan to address data privacy given sensitive information being posted in chatbot. The project is ambitious for 2 years (qualitative study, design chatbot (prototype made) and pilot study. Focus on rural may decrease generalizability. Does aim 1 need 36 participants for theme saturation? Or can that be decreased. Wil this be women only? Should men be included? The PCCC scale is validated and better than past contraceptive scales but has some flaws in terms of being designed for use immediately after a visit (not necessarily reliability longer after the visit), demographic disparities, and top-box scoring. It also doesn't really capture contraceptive coercion - or how contraceptive information is incorporated by patients (which was a part of the premise regarding misinformation and health literacy in rural populations).

4. Mentors

Instructions Provided to Applicants: *Candidates can include up to three mentors as part of this application. However, one must be designated as the primary mentor. The primary mentor is directly accountable for scholar progress and should help the scholar manage the broader team. For each mentor, we will ask for the following information:*

- *Mentor information, including position and past mentor training*
- *Recent NIH Biosketch, uploaded directly into the application portal*
- *Information about mentoring experience*

A letter of support from the applicant's primary mentor is required. ***Applicants can submit up to two additional letters (e.g., from other mentors and collaborators), though these are optional.***

Guiding Questions for Reviewers:

- Does the mentor team make sense for the proposed research?
- As a team, do the proposed mentors have sufficient mentorship experience?

- Does the primary mentor's letter of support demonstrate sufficient commitment to the applicant's development?
- Is sufficient rationale given for each mentor in alignment with the career development plan?

Score: 1: Exceptionally strong with essentially no weaknesses

Strengths: *Redacted for all applications*

Weaknesses: *Redacted for all applications*

5. Institutional Commitment to Candidate

Instructions Provided to Applicants: *We require a letter of institutional support (department chair or division chief). This should include a commitment of 75% effort to the K12 program and salary co-funding, if needed.*

Guiding Questions for Reviewers:

- Do you feel the applicant has institutional/departmental support that will complement the K12?
- Does the candidate have a clear track to faculty position if they do not have one already?

Score: 1: Exceptionally strong with essentially no weaknesses

Strengths: *Redacted for all applications*

Weaknesses: *Redacted for all applications*
