# Supplementary material for: Trust-Centered Design and Feasibility Evaluation of an AI-Enabled Conversational Health Tool for Sexual and Reproductive Health Among Rural Young Adults: Protocol for a Mixed Methods Study
Source: JMIR Res Protoc. 2026 Aug 6;15:e97653. doi: 10.2196/97653 (PMC13446796; doi:10.2196/97653)
Supplement: Supplementary file 2 [file resprot-v15-e97653-s003.pdf]

UNC BIRCWH REVIEW TEMPLATE

Reviewer Name: Reviewer 2

Candidate Name: Kandyce Brennan

Scoring Criteria

- Please use a separate template for each candidate you review.
- ***We will remove your name from this review prior to sharing it with the candidate.***
- We will use the NIH 9-point scale (no decimals).
    1. Exceptionally strong with essentially no weaknesses
    2. Extremely strong with negligible weaknesses
    3. Very strong with only some minor weaknesses [a]
    4. Strong, but with numerous minor weaknesses
    5. Strong, but with at least one moderate weakness [b]
    6. Some strengths, but also some moderate weaknesses
    7. Some strengths, but with at least one major weakness [c]
    8. A few strengths and a few major weaknesses
    9. Very few strengths and numerous major weaknesses
- Weaknesses are defined as follows:
    a. Minor weakness: An easily addressable weakness that does not substantially lessen impact
    b. Moderate weakness: A weakness that lessens impact
    c. Major weakness: A weakness that severely limits impact

Overall Impact

Provide an overall impact score reflecting your assessment of the likelihood for the candidate to initiate a strong research program, in consideration of the five scored review criteria (below) and the following program priorities:

- **BIRCWH program priorities:** Mentor and train early career investigators to become independent interdisciplinary translational science researchers who study high priority areas in women's health, explore biological sex influences on health and disease, and incorporate research strategies to diminish health disparities for women across the lifespan.

Overall Impact Score: 2: Extremely strong with negligible weaknesses

Overall Impact Summary: Dr. Brennan is a promising early-stage investigator whose trajectory aligns well with the UNC BIRCWH program's mission. She brings a compelling blend of clinical expertise in midwifery, documented productivity in AI-enabled sexual and reproductive health (SRH) research, and a focused commitment to addressing disparities affecting rural pregnant-capable young adults. Her application demonstrates strong research traction, including peer-reviewed publications, funded pilot grants, and early feasibility work on SARHAchat. Dr. Brennan proposes training in mixed methods, human-centered design, and implementation science, central to her research trajectory. The research project is innovative, highly responsive to translational gaps in trustworthy AI for SRH, and appropriately scoped for a K transition to an R award. The mentorship team is robust, interdisciplinary, and well-matched to both training and scientific needs. Institutional support is clearly articulated. Weaknesses are minor and primarily related to the breadth of training activities,

which will require prioritization and refinement. Overall, the likelihood that this candidate will transition to independence as a funded translational women's health scientist is very high.

Recommendation: Discuss in study section.

Scored Review Criteria

Please provide a separate score for each criterion.

1. Candidate Background and Research Training

Instructions Provided to Applicants: *A recent Biosketch should be uploaded into the application portal. Use updated format dated September 2024.*

Guiding Questions for Reviewers:

- Evaluate the developmental stage of the candidate. Do they have some research "traction" while still having room to benefit from additional focused training?
- Do the candidate's career goals align with the K12 program?

Score: 2: Extremely strong with negligible weaknesses

Strengths: Strong clinical foundation as a certified nurse-midwife with extensive experience in rural and underserved settings. This lived expertise meaningfully enhances her translational perspective. Clear and coherent research trajectory progressing from clinical observations to dissertation research on SRH access to AI-enabled digital health innovation. Early evidence of research productivity, including peer-reviewed publications, an in-press manuscript, and funded pilot work through Society of Family Planning, UNC Data Science and Society, and School of Nursing awards. Demonstrated capacity to lead interdisciplinary projects, including development and feasibility testing of SARHAchat. The biosketch reflects a strong track record of scholarly products appropriate for her stage. Clear gap between current skillset and future goals, validating the need for structured K-level training.

Weaknesses: (Minor) Dr. Brennan's formal methodological training in mixed methods and advanced implementation science remains nascent, but the plan adequately addresses these gaps.

2. Candidate Career Development Plan

Instructions Provided to Applicants: *Clearly identify your training goals during the K12 program and explain how each goal aligns with your current research project and long-term career objectives. Be specific in describing how the proposed training activities will directly support your transition to becoming an independent researcher. Where appropriate, including expected timelines and completion dates for specific activities.*

Guiding Questions for Reviewers:

- Is there sufficient rationale for training?
- Does the training plan align with long term goals, i.e. learning skills that will serve to define a new niche for the candidate?
- Does the career development plan include content on community engagement and/or health disparities training?

Score: 3: Very strong with only some minor weaknesses

Strengths: Explicit, well-justified training goals that map cleanly onto the research aims and long-term objective of becoming an independent investigator. Comprehensive plan that integrates mixed methods, HCD, and implementation science; these domains are essential to her intended research interests. Strong and thoughtfully

designed timeline with specific workshops, coursework, mentored experiences, and manuscript plans. Appropriate incorporation of community engagement and disparities-focused training. Professional development elements (grant-writing, conference engagement, networking) are practical and forward-looking.

Weaknesses: (Minor) Plan is ambitious and to an extent unwieldy, but it is achievable. It will require prioritization such that the training does not seem fragmented and runs off-course. (Minor) The training plan could outline some contingency options if certain training components (e.g., elective courses) become unavailable.

3. Proposed Research Project

Instructions Provided to Applicants: *This project serves as the scholar's primary research effort. It is expected to generate most or all of the preliminary data needed for an individual K- or R-equivalent grant submission by the end of the second year of support. This plan should address key components such as the significance, innovation, and approach of the proposed project. Organize this section according to the following subsections:*

- *I. Specific Aims (maximum 1 page)*
- *II. Research Strategy (maximum 4 pages):*
    - *a. Significance: Describe the importance of the problem, how it will advance the field, and the potential impact of your research.*
    - *b. Innovation: Explain how your research challenges existing paradigms or develops novel technologies or approaches.*
    - *c. Approach: Detail the experiments you will conduct to achieve each aim organized around those aims. Include your rationale for the hypothesis, expected outcomes, and alternative approaches.*

Guiding Questions for Reviewers:

- Does the research plan describe the significance and innovation potential of the research to advance women's health?
- Does the research plan serve as a foundation for future research?
- Does the research plan describe a project that will generate preliminary data for the next step of grant submission within the 2-to-3-year proposed timeframe?
- Does the scope of the research project align with the proposed timeline?

Score: 4: Strong, but with numerous minor weaknesses

Strengths: The project addresses a timely and high-priority problem. The significance of the proposed research is strong. The proposed research directly targets health disparity, rural women's health, and SRH access aligning with UNC BIRCWH priorities. The innovation is clear with an explicit focus on trust-centered HCD for conversational AI, community co-design, and implementation strategies for AI-enabled SRH decision support. The preliminary data from SARHAchat is compelling and establishes feasibility while revealing gaps suitable for refinement. The three aims form a tight, logical progression, which includes qualitative inquiry, HCD co-design, and a feasibility pilot. Each aim is geared toward a future R award. Research strategy is detailed, methodologically rigorous, and leverages validated measures and RE-AIM for implementation outcomes. Scope is realistically sized for a K12 with manageable sample sizes, structured timelines, and reliance on existing infrastructure and mentorship support.

Weaknesses: (Minor) The project spans multiple methodological domains that can be challenging to execute at the same scope and depth. Prioritization will be essential. (Minor) Social media recruitment strategies for rural populations can be unpredictable. There is a need to outline (alternate) contingency plans. (Minor) The role of community partners can be highlighted more. CBPR necessitates engagement of communities on equal footing from conceptualization to dissemination. A deeper engagement with community partners could be better elaborated in the proposed plan and execution.

4. Mentors

Instructions Provided to Applicants: *Candidates can include up to three mentors as part of this application. However, one must be designated as the primary mentor. The primary mentor is directly accountable for scholar progress and should help the scholar manage the broader team. For each mentor, we will ask for the following information:*

- *Mentor information, including position and past mentor training*
- *Recent NIH Biosketch, uploaded directly into the application portal*
- *Information about mentoring experience*

A letter of support from the applicant's primary mentor is required. ***Applicants can submit up to two additional letters (e.g., from other mentors and collaborators), though these are optional.***

Guiding Questions for Reviewers:

- Does the mentor team make sense for the proposed research?
- As a team, do the proposed mentors have sufficient mentorship experience?
- Does the primary mentor's letter of support demonstrate sufficient commitment to the applicant's development?
- Is sufficient rationale given for each mentor in alignment with the career development plan?

Score: 1: Exceptionally strong with essentially no weaknesses

Strengths: *Redacted for all applications*

Weaknesses: *Redacted for all applications*

5. Institutional Commitment to Candidate

Instructions Provided to Applicants: *We require a letter of institutional support (department chair or division chief). This should include a commitment of 75% effort to the K12 program and salary co-funding, if needed.*

Guiding Questions for Reviewers:

- Do you feel the applicant has institutional/departmental support that will complement the K12?
- Does the candidate have a clear track to faculty position if they do not have one already?

Score: 1: Exceptionally strong with essentially no weaknesses

Strengths: *Redacted for all applications*

Weaknesses: *Redacted for all applications*
