# Supplementary material for: Trust-Centered Design and Feasibility Evaluation of an AI-Enabled Conversational Health Tool for Sexual and Reproductive Health Among Rural Young Adults: Protocol for a Mixed Methods Study
Source: JMIR Res Protoc. 2026 Aug 6;15:e97653. doi: 10.2196/97653 (PMC13446796; doi:10.2196/97653)
Supplement: Supplementary file 3 [file resprot-v15-e97653-s001.docx]

**Appendix 1. RE-AIM Joint Display**

| **RE-AIM Domain (measure)** | **Quantitative Result (vs. pre-specified threshold)** | **Qualitative Finding (explanatory exit interviews)** | **Integrated Meta-Inference (fit type → implication)** |
| --- | --- | --- | --- |
| **Reach (enrollment & consent rates)** |  |  |  |
| **Effectiveness (Decision Self-Efficacy Scale [DSES]; adapted General Help-Seeking Questionnaire [GHSQ])** |  |  |  |
| **Adoption / Engagement (session completion; backend exchanges)** |  |  |  |
| ***Implementation*** | | | |
| **Usability (BUS-11)** |  |  |  |
| **Acceptability (AIM)** |  |  |  |
| **Appropriateness (IAM)** |  |  |  |
| **Trust (TPA)** |  |  |  |
| **Retention (T1 completion)** |  |  |  |
| **Maintenance (tool uptime; error/failure rate)** |  |  |  |

Fit type denotes the relationship between the quantitative and qualitative findings for each domain: confirmation (the two data strands agree), expansion (the qualitative data add nuance or explain variation the quantitative result alone does not capture), or discordance (the two strands diverge and require reconciliation), per Fetters, Curry & Creswell (2013). Meta-inferences will inform the pre-specified progression decision and distinguish modifiable design-level barriers from structural ones.

Abbreviations: AIM, Acceptability of Intervention Measure; BUS-11, Bot Usability Scale; IAM, Intervention Appropriateness Measure; RE-AIM, Reach, Effectiveness, Adoption, Implementation, Maintenance; T1, post-interaction timepoint; TPA, Trust between People and Automation scale.
